# Poor birth weight recovery among low birth weight/preterm infants following hospital discharge in Kampala, Uganda

**DOI:** 10.1186/1471-2393-12-1

**Published:** 2012-01-09

**Authors:** Flavia B Namiiro, Jamiir Mugalu, Ryan M McAdams, Grace Ndeezi

**Affiliations:** 1 Department of Pediatrics, Mulago National Referral Hospital, Kampala, Uganda; 2 Division of Neonatology, University of Washington, Seattle, Washington, USA; 3 Department of Pediatrics, Makerere University, College of Health Sciences (MakCHS), Kampala, Uganda

## Abstract

**Background:**

Healthy infants typically regain their birth weight by 21 days of age; however, failure to do so may be due to medical, nutritional or environmental factors. Globally, the incidence of low birth weight deliveries is high, but few studies have assessed the postnatal weight changes in this category of infants, especially in Africa. The aim was to determine what proportion of LBW infants had not regained their birth weight by 21 days of age after discharge from the Special Care Unit of Mulago hospital, Kampala.

**Methods:**

A cross sectional study was conducted assessing weight recovery of 235 LBW infants attending the Kangaroo Clinic in the Special Care Unit of Mulago Hospital between January and April 2010. Infants aged 21 days with a documented birth weight and whose mothers gave consent to participate were included in the study. Baseline information was collected on demographic characteristics, history on pregnancy, delivery and postnatal outcome through interviews. Pertinent infant information like gestation age, diagnosis and management was obtained from the medical records and summarized in the case report forms.

**Results:**

Of the 235 LBW infants, 113 (48.1%) had not regained their birth weight by 21 days. Duration of hospitalization for more than 7 days (AOR: 4.2; 95% CI: 2.3 - 7.6; p value < 0.001) and initiation of the first feed after 48 hours (AOR: 1.9; 95% CI 1.1 - 3.4 p value 0.034) were independently associated with failure to regain birth weight. Maternal factors and the infant's physical examination findings were not significantly associated with failure to regain birth weight by 21 days of age.

**Conclusion:**

Failure to regain birth weight among LBW infants by 21 days of age is a common problem in Mulago Hospital occurring in almost half of the neonates attending the Kangaroo clinic. Currently, the burden of morbidity in this group of high-risk infants is undetected and unaddressed in many developing countries. Measures for consideration to improve care of these infants would include; discharge after regaining birth weight and use of total parenteral nutrition. However, due to the pressure of space, keeping the baby and mother is not feasible at the moment hence the need for a strong community system to boost care of the infant. Close networking with support groups within the child's environment could help alleviate this problem.

## Background

The prevalence of low birth weight (LBW) deliveries is higher in developing countries than developed countries and is associated with increased risks of poor health outcomes[1]. The World Health Organization (WHO) defines LBW as the weight of live born infants less than 2,500 grams. The condition can be due to premature birth or intrauterine growth restriction or both[1,2]. Low birth weight, which is approximately equivalent to preterm birth, is an important predictor of infant death within 28 days of birth. It is estimated that LBW infants are approximately 13 times more likely to die than heavier babies[3]. Countries, such as Uganda, with higher rates of preterm delivery have higher rates of infant mortality[3,4].

More than 20 million infants worldwide are born with LBW with the majority (95.6%) born in developing countries, mainly South Asia and Sub Saharan Africa[1,2]. Globally, there has been increasing awareness of specialized care required for LBW infants due to advances in Neonatal-Perinatal medicine. However, instituting this specialized care is still a challenge in developing countries[5]. Almost 4 million deaths occur in the first month of life especially among the LBW infants[6]. Although in many African countries birth weight is rarely recorded, in the sub-Saharan region estimates of LBW are around 14.3 percent, which is almost double the frequency in European countries[7,8]. Saving newborn life is necessary in order to improve child health, reduce infant mortality rate and to attain Millennium Development goal IV in many developing countries. Providing extra support to LBW infants regarding feeding, warmth and growth monitoring has great potential to reduce neonatal mortality rate and promote survival with good quality of life[7].

Growth assessment in the neonatal period is determined by changes in anthropometric measurements and consistent weight gain is a valuable guide indicating adequate growth[9]. The change in weight during the neonatal period of LBW infants is characterized by an initial loss of approximately 8-15% of the birth weight in the first 7 days following delivery and thereafter recovery occurs within 10 to 21 days [10,11]. Postnatal weight loss in the early neonatal period is greater in very LBW (weigh 1000-1500 grams) and extreme LBW (weigh less than 1000 grams) infants, although considerable variation occurs among individual infants. Growth delay or failure to regain birth weight may occur due to prevailing postnatal care practices and/or various factors, which may be medically, nutritionally or environmentally related[11]. It has been reported that infants with extreme low birth weight are more susceptible to all of the possible complications of premature birth, both in the immediate neonatal period and after discharge from the nursery[12].

In Uganda, one out of seven newborns is LBW and requires specialized care. In the Special Care Unit (SCU) of Mulago Hospital in Kampala, the high prevalence of LBW infants poses constraints on available resources. Infants are often discharged early and followed up in the Kangaroo Clinic. Unfortunately, many mothers do not adhere to the recommended follow-up schedule due to poverty-associated reasons. This lack of timely follow-up frequently results in delayed identification of neonatal problems such as poor weight gain[6].

Maternal factors that have been reported to affect postnatal outcome include maternal age, level of education, socio-economic status and illness[13]. Neonatal issues that may influence postnatal growth include prematurity, which may cause feeding problems (e.g, failure to latch on the breast), respiratory distress, hypothermia, necrotizing enterocolitis, and asphyxia[14,15]. In the SCU of Mulago Hospital, there is limited space for mothers to room-in in relation to the number of LBW infants admitted. This affects mother/baby feeding and bonding, as well as the quality of services given, such as thermal control and parental counseling on infant care. Typically, there is no discharge planning or home assessment done before a LBW infant leaves the hospital. Additionally, the amount of social support a mother and infant will receive from their family and community is usually not clear. In light of these challenges, we describe weight changes of LBW infants followed up in a Kangaroo clinic following discharge from the SCU at Mulago Hospital in Kampala, Uganda's capital.

## Methods

### Study site

This cross sectional study was conducted in the Neonatal SCU of Mulago Hospital between January and April 2010. Mulago hospital is the National Referral Hospital for Uganda and teaching hospital for Makerere University. The hospital has a total bed capacity of 1,500 and an annual inpatient turnover of 120,000. Special Care Unit receives babies from throughout Uganda, but primarily serves a poor urban population. At full capacity, SCU has 59 incubators/cots, with a provision for rooming-in of 6 adult beds. The mothers stay on the postnatal ward and check on their infants at two hourly intervals both day and night. On average, the unit admits three LBW infants daily. The majority of infant's are fed on breast milk, however, complementary feeding is sometimes done for the mothers who fail to produce breast milk, typically using Nan 1 infant formula (Nestle) and cow's milk.

The SCU runs a cost-free Kangaroo clinic for LBW/preterm infants following discharge. Infants are seen twice a week, every Tuesday and Friday, during which their wellbeing is assessed and parents' concerns on feeding or other medical issues are addressed. Infants are followed up regularly until they attain a weight of 2,500 grams and then are subsequently followed in the preterm clinic, which they attend at 1-3 months' intervals until the age of 2 years. On average, 30 to 45 neonates are seen in the Kangaroo clinic each day and the interval between subsequent visits depends on how well the baby is thriving post-discharge with consideration for readmission when necessary.

### Study instrument

The study instrument was a structured, pre-coded case report form, written in English, and administered by the Principal investigator (FN) or the research assistant. The case report was administered in the language best understood by the mothers of the study participants. We used both qualitative and quantitative methods of collecting data. Interviews were semi-structured, using both open-ended and closed-ended questions. Pertinent patient information such as birth weight, gestational age, and interventions done during admission were obtained from the medical records and summarized in the case report forms. The infant's gestational age was estimated by the admitting doctor (the majority were pediatric residents) in the first 24 hours of age using the New Ballard Score[16]. All LBW infants were weighed twice on admission to SCU and measurements were recorded in the medical records. The same digital weighing scale, accurate to 5 grams and calibrated before each measurement, was used during the current study. All weight measurements were performed with the infants naked.

Mothers were advised at the time of discharge to attend the Kangaroo clinic on the day closest to the 21st day of age for each infant. However, based on the baby's stability at the time of discharge, the physician on duty determined the follow-up dates. Discharge criteria from the SCU required that the baby had to be tolerating at least 10 mls per feed, the mother had to demonstrate knowledge and competency with feeding, especially if the baby was to go home with the nasal gastric tube in situ, and the baby had to be clinically stable (e.g., normal vital signs and no life threatening medical issues). Upon discharge, routine information was provided by SCU staff to all mothers on proper feeding, thermoregulation (especially using the skin-to-skin method for the very small infants), immunizations, and need for follow-up after discharge.

### Study participants

**Figure 1:**
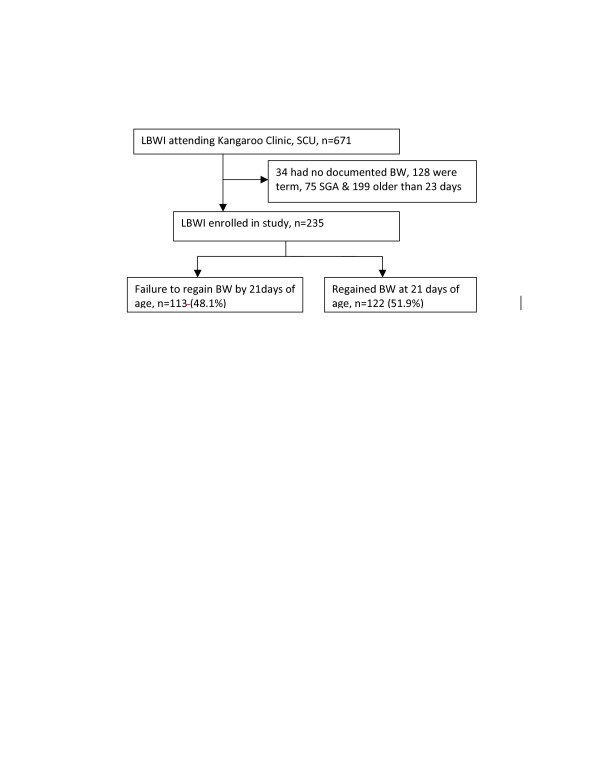
**Study schema**. Abbreviations; LBWI: Low birth weight Infants, SCU: Special care Unit, BWT: Birth weight, SGA: Small for gestation age.

All low birth weight neonates seen in the clinic between January and April 2010 with a documented birth weight and aged 21 ± 2 days were eligible. Relevant details regarding gestational age, discharge weight, diagnoses and management practices performed during admission were obtained from the medical records. Infants were excluded from the study if they were small for gestation age, defined as birth weight below the 10th percentile for the gestational age (Figure 1). At three weeks of age, baseline information was collected on household demographic characteristics and history of the pregnancy, delivery, and immediate postnatal period for each subject. Twins and triplets were treated as independent observations. Anthropometric measurements (weight, length and head circumference) and physical examination were carried out and recorded. Eligible participants were consecutively enrolled until the desired sample size was assumed. Neonates were recruited every Tuesday and Friday, between 0800 and 1400 hours. The sample size was 235 infants and the magnitude of desired precision was 80%. The sample size was calculated using the Kish Leslie formula for 50% prevalence and adjusted from a finite population of 513 infants seen in the clinic between August and December 2009.

### Ethical considerations

Permission to conduct the study was granted by Makerere University College of Health Sciences and by the Mulago Hospital Research and ethics Committee. Informed verbal consent was obtained from each caregiver. Detailed information on study procedures and purpose were explained to the caretakers. The study used numbers for identification of participants and the responses of the mothers were kept confidential.

### Data management and statistical analysis

Raw data from the case report form was cross-checked for completeness, cleaned and entered into a computerized database using Epi Data 3.1. Statistical analysis was done using STATA version 10 software (College Station, Texas, USA). The prevalence of failure to regain birth weight among LBW infants was calculated by dividing the number of infants who had not regained their birth weight at 21 days of age by the total number of infants enrolled in the study. Categorical data to test for presence of association between failure to regain birth weight and the different variables mentioned above was analyzed using Fischers exact test. Students t test was used for continuous variables. The strength of association between the factors and failure to regain birth weight among the infants studied was determined using odds ratios and confidence intervals. All factors considered at bivariate analysis were entered into a logistic regression model. Factors with p value ≤ 0.2 at bivariate analysis were subjected to the backward and forward stepwise methods at multivariate analysis to determine the factors associated with failure to regain birth weight. P values of < 0.05 were considered significant and confidence interval of 95% was used.

## Results

A total of 671 infants were seen in the Kangaroo clinic during the study period: 235 infants met the eligibility criteria and were enrolled consecutively on each clinic day. One hundred and twenty eight infants were term, 75 were small for gestation age, 34 had no documented birth weights and 199 infants were older than 23 days. The characteristics of the study participants at birth and at 21 days of age are summarized in Table 1

**Table 1:** Characteristics of study participants at 21 days

Variable	All infants N = 235	%	Regained BW N = 122	%	Did not regain BW N = 113	%
**Sex**						
Male	121	51.5	68	55.7	53	46.9
Female	114	48.5	54	44.3	60	53.1
**Gestation age(weeks)**						
**≤ 32**	88	37.4	39	32.0	49	43.4
**> 32**	147	62.6	83	68.0	64	56.6
**Birth type**						
Single	171	72.8	93	76.2	78	69.0
Multiple	64	27.2	29	23.8	35	31.0
**Weight at birth**						
**≤ **1500 g	88	37.4	39	32.0	49	43.4
> 1500**g**	147	62.6	83	68.0	64	56.6
**Initiation of first feed**						
**> 4**8 hours	90	38.3	34	27.9	56	49.6
≤ 48 hours	145	61.7	88	72.1	57	50.4
**Mode of feeding**						
Exclusive breastfeeding						
Yes	219	93.2	116	95.1	103	91.2
No	16	6.8	6	4.9	10	8.8
Complementary feeding						
Yes	20	8.5	9	7.4	11	9.7
No	215	91.5	113	92.6	102	90.3
Gavage feeding						
Yes	153	65.1	71	41.8	82	27.4
No	82	34.9	51	58.2	31	72.6
**Duration of hospital stay**						
≤ 7 days	150	63.8	98	80.3	52	46.0
> 7 days	85	36.2	24	19.7	61	54.0

Although 37.4% (88/235) of infants had a birth weight of ≤ 1500 grams, the number of infants with measured weights of ≤ 1500 grams increased at 21 days to 42.6% (100/235). Only four infants weighed less than 1000 grams and they had not regained weight by 21 days of age. The mean gestation age for the participants was 32.0 weeks.

Sixty-four infants in the study were multiples with 2 sets of triplets and the rest twins. Death occurred in 39% (24/64) of the multiples by the time of enrolment in the study. One set of triplets had a gestation age of 35 weeks and all three weighed between 1500 grams and 2000 grams, with 2 regaining their birth weight by 21 days of age. Two of the second set of triplets died and only one lived to 21 days of age, with a gestational age of 32 weeks, weighing 1260 grams at birth and 1340 grams at 21 days of age.

Table 2 shows the socio-demographic characteristics of the mothers (191 mothers were enrolled, but data analysis was done for each mother/infant pair). Eighty-two (34.9%) of the mothers were aged 20 years or less and 87 (37%) attained post primary education level.

**Table 2:** Socio-demographic characteristics of infants and their mothers

Variable	Regained BW N = 122	%	Did not Regain BW N = 113	%
**Maternal Age (years)**				
**≤ **20	42	51.2	40	48.8
21-25	47	56.6	36	43.4
26-29	23	47.9	25	52.1
≥ 30	10	45.4	12	54.6
**Mode of delivery**				
SVD	101	82.8	103	91.2
C/S	21	17.2	10	8.8
**Marital status**				
Married	102	54.8	84	45.2
Single	20	40.8	29	59.2
**Education level**				
None/primary	75	61.5	73	64.6
Secondary +	47	38.5	40	35.4
**Mother's occupation**				
None	14	11.4	12	10.6
House wife	54	44.3	47	41.6
Other*	54	44.3	54	47.8
				
ANC Attendance**				
Yes	110	90.2	97	85.2
No	12	9.8	16	14.2
**Illness in pregnancy**				
Yes	53	43.4	47	41.6
No	69	56.6	66	58.4
**HIV status**				
**P**ositive	10	8.2	16	14.2
Negative	112	91.8	97	85.8
**Father's education**				
None***	35	28.7	21	37.5
Primary	12	9.8	8	40
Secondary	52	42.6	56	51.8
Tertiary	23	18.9	28	54.9

*Any form of money generating activity;**ANC, antenatal care, attendance during pregnancy;***No formal education or unknown. Abbreviations: BW, birth weight

The maternal factors and physical examination were not found significantly associated with failure to regain birth weight in the first 21 days of age as shown in table 3.

**Table 3:** Factors associated failure to regain birth weight

Variable	N = 113	%	COR	95%CI	pvalue
**Gestation age (in weeks)**					
> 32	64	43.4	1.63	0.92-2.90	0.08
≤ 32	49	56.6	1.00		
**Birth type**					
Singleton	78	69	1.44	0.78-2.67	0.242
Multiple	35	31	1.00		
**Initiation of first feed**					
≤ 48 hours	43	37.8	2.54	1.43-4.53	0.001*
**>**48 hours	70	62.2	1.00		
**Mode of feeding**					
Exclusive breastfeeding					
Yes	103	91.2	1.88	0.59-6.49	0.302
No	10	8.8	1.00		
Complementary feeding					
No	102	90.39.7	1.34	0.48-3.82	0.642
Yes	11		1.00		
Gavage feeding					
Yes	82	72.6	1.9	1.06-3.42	0.028*
No	31	27.4	1.00		
**Duration of hospital stay**					
≤ **7 **days	52	46	4.79	2.57-8.95	0.001*
> 7 days	61	54	1.00		

* p value < 0.05 COR- Crude Odds Ratio

### Factors associated with failure to regain birth weight

From the logistic regression model (Table 4), hospital stay of more than 7 days (p value: 0.001) and initiation of first feed of more than 48 hours (0.034) were the significant factors that contributed to failure to regain birth weight among the study participants.

**Table 4:** Logistic regression for factors associated with failure to regain birth weight

Variable	COR*	95% CI	AOR*	95% CI	p value
Time of initiation of first feed	1.87	1.05-3.35	1.91	1.07-3.40	**0.034**
Gavage feeding	1.9	1.06-3.42	1.2	0.65-2.20	0.561
Number of days of hospital stay	4.11	2.27-7.44	4.17	2.30-7.55	**0.001**

COR* Denotes Crude Odds ratio AOR* Denotes Adjusted Odds ratio

## Discussion

The current study, reflective of a predominantly low-income urban maternal and neonatal population in Uganda, provides important insights about neonatal risk factors contributing to post-discharge growth failure in LBW infants. Almost half of the LBW infants seen in the Kangaroo Clinic at Mulago Hospital, Kampala, had not regained their birth weight by 21 days of age. This is surprising since LBW infants should typically regain their birth weight within 10 to 21 days of age[17]. Due to the limited resources and institutional restrictions, one may speculate that infants in the study would have received more personalized care promoting improved growth while at home; however, this does appear to be the case.

The large number of LBW infants in the SCU poses constraints on the available resources, which in turn may hinder optimal care encouraging good growth. The limited resources endorse early discharge of infants from the SCU with follow-up in the Kangaroo clinic. The potential success of this system is contingent on adherence to the scheduled appointments. Lack of follow-up precludes to failure to identify neonatal complications in a timely manner, which may have consequences on the infant's wellbeing, both short- and long-term. A limitation of our study is the absence of information on those who did not return to the Kangaroo clinic for follow-up. We were not able to differentiate non-participation versus non-attendance at the Kangaroo Clinic because the number of neonates who were expected to return to the clinic during the study period was not recorded. It is not clear if our data are more reflective of a follow-up bias population skewed toward infants with poor post-discharge weight gain or whether the infants in our study represent the typical weight gain pattern in LBW infants in similar low-income settings.

It appears that early discharge was detrimental to proper post-natal growth in many of the study participants. Although a more prolonged hospital stay offers an appealing, albeit costly, alternative for supporting proper weight gain and neonatal care in low-income countries, this option is not currently feasible in settings similar to Uganda. The factors that significantly contributed to failure to regain birth weight among the LBW infants were hospital stay of more than 7 days and initiation of first feed more than 48 hours. We did not explore in detail the reason for long hospital stays among our study infants, however prolonged stay due to medical illness may be fatal to growth as opposed to stay for nutritional support. Feeding in the first one hour of life (WHO recommendation) may not be possible in many of the LBW infants and other institutional setups, like the initial separation of the mother and baby may contribute to the delay. Although there have been documented cost-effective interventions in neonatal care, few reach preterm infants in low-income countries[6,18]. Further research is needed to assess care practices given to the infants while at home in order to better understand any factors that may be contributing to poor weight gain. The need for simple interventions that can be implemented following discharge is particularly great since keeping infants hospitalized until they attain weights of 2000-2500 grams is not possible due to lack of space. Simple interventions to improve infant care in the community may include women support groups, family members and the village health teams to encourage infant feeding, skin-to-skin care, and community healthcare worker assessments to identify and treat neonatal sepsis. This has been demonstrated to be feasible in some low resource settings in Asia and has been proved successful[18].

Although it was not one of our study objectives, we found that among the multiple infants, 39% (25/64) had lost their siblings by 21 days of age. This is a high prevalence and concurs with other findings of risks, morbidity and mortality associated with preterm multiple births[19]. Special attention should be given to these high-risk infants regarding discharge planning, parental counseling, and follow-up evaluations to help prevent adverse outcomes. Future studies are needed to determine the etiology of this increased mortality in LBW multiple infants discharged from the SCU

This study had a number of limitations. It did not include a matched control group to which outcomes could have been compared. We were not able to use dry weight since it was not feasible to withhold feeds from infants for more than 2 hours and their reporting time to the clinic varied. The estimated gestational ages may have not always been accurate since they were based off maternal history of last menstrual period and Ballard scores. In the antenatal care clinic, fetal ultrasonography, the most accurate technique for estimating gestational age, is not routinely done and costs a fee, which most mothers cannot afford. We had no control over timing of discharge and scheduling of follow-up dates. Ideally, all infants would have been seen at 21 days of age: however, this could not be done due partly to logistic reasons, post-discharge state of the infant, and caretaker's decision on when to return to the clinic. Many infants were excluded due to post-dates and this could have affected our results. Since all recorded information was obtained from the mother and the available medical records, recall bias and incomplete documentation respectively, may have affected our results. Additionally, maternal nutritional status and health was not assessed, which may have impacted the growth of the infants in the study.

## Conclusion

Failure to regain birth weight among low birth weight infants by 21 days of age is a common problem in Mulago Hospital occurring in almost half of the neonates attending the Kangaroo clinic. Currently, the burden of morbidity in this group of high-risk infants is undetected and unaddressed in many developing countries. Measures for consideration to improve care of these infants would include; discharge after regaining birth weight and use of total parenteral nutrition. However, due to the pressure for space, keeping the baby and mother is not feasible at the moment hence the need for a strong community system to boost care of the infant. Close networking with support groups within the child's environment could help improve growth. There is a need for evaluation of simple, cost effective interventions in the community settings prior to discharge of the LBW infants.

## Competing interests

The authors declare that they have no competing interests.

## Contributors

FN, JM & GN contributed to the design of the study and assisted with data analysis. FN coordinated the study and supervised the enrolment of patients. All authors contributed to the interpretation of data, preparation of the manuscript and approved the final version.

## Pre-publication history

The pre-publication history for this paper can be accessed here:

http://www.biomedcentral.com/1471-2393/12/1/prepub
